# Trends in alcohol-related admissions to hospital by age, sex and socioeconomic deprivation in England, 2002/03 to 2013/14

**DOI:** 10.1186/s12889-017-4265-0

**Published:** 2017-05-08

**Authors:** Mark A Green, Mark Strong, Lucy Conway, Ravi Maheswaran

**Affiliations:** 1 Department of Geography & Planning, University of Liverpool, Liverpool, UK; 2 Public Health GIS Unit, School of Health and Related Research (ScHARR), University of Sheffield, Regent Court, 30 Regent Street, Sheffield, S1 4DA UK; 3 Public Health England, London, UK

**Keywords:** Alcohol-related Disorders, Alcohol-induced Disorders, Trends, Alcoholic liver diseases, Population characteristics, Poverty, England

## Abstract

**Background:**

Prevalence of alcohol-related harms in England are among the highest in Europe and represents an important policy issue. Understanding how alcohol-related trends vary by demographic factors is important for informing policy debates. The aim of our study was to examine trends in alcohol-related admissions to hospital in England, with a focus on variations by sex, age and socioeconomic deprivation.

**Methods:**

We used data on hospital admissions for England for the financial years 2002/03 to 2013/14. Our four main outcome variables were acute and chronic conditions wholly and partially attributable to alcohol consumption. We also looked at four specific conditions wholly attributable to alcohol. Socioeconomic deprivation was measured using the English Indices of Deprivation of a patient’s residence (categorised by quintile). We calculated crude rates, age-specific rates (visualised by Lexis plots) and directly standardised rates by deprivation category, separately for males and females.

**Results:**

Total admissions for all alcohol-attributable admissions increased from 201,398 in 2002/03 to 303,716 in 2013/14. The relative increase of these admissions was larger than compared to non-alcohol attributable admissions. Acute admissions wholly attributable to alcohol had the largest relative increase of our outcome measures, and displayed a bimodal distribution with higher rates in adolescence/young adults and middle age. Chronic conditions wholly attributable to alcohol were concentrated in middle age (particularly males). While admission rates were generally higher for males, females had higher rates of hospitalisations due to ‘Intentional self-poisoning due to alcohol’. We also found evidence of wide social inequalities by level of deprivation, which were wider for men than compared to women across all of our outcome measures other than ‘Intentional self-poisoning due to alcohol’.

**Conclusions:**

Our study expands the evidence base to help understand population level trends in alcohol-related admissions by age, sex and socioeconomic deprivation. There have been increasing hospital admissions attributable to alcohol between 2002/03 and 2013/14, particularly concentrated in middle aged males and deprived areas. However, the increase in young females being admitted for ‘Intentional self-poisoning due to alcohol’ raises additional concerns.

**Electronic supplementary material:**

The online version of this article (doi:10.1186/s12889-017-4265-0) contains supplementary material, which is available to authorized users.

## Background

The consumption of alcohol is associated with a number of acute and chronic health conditions [1–4]. Alcohol consumption is common in England, with latest estimates suggesting that 79% of individuals consume alcohol and 1.6 million individuals (9% of adult males and 4% of adult females) may have some level of dependence on alcohol [5, 6]. The combination of these factors places considerable burden on health services, and is estimated to cost the National Health Service (NHS) £3.5 billion per year [7]. This figure ignores the wider costs to society of alcohol use, such as crime and lost productivity, which are estimated at £21.5 billion [6]. Alcohol is also one of the main contributors to social inequalities [4, 8–12]. Given that many alcohol-related issues are lifestyle induced (i.e. a result of excessive consumption), they are potentially preventable and therefore an opportunity for policy to target. As such, these issues have placed alcohol as a pressing and important public health concern.

Whilst risk of hospital admission for alcohol-related conditions has been demonstrated to be associated with age, sex and socioeconomic deprivation [13–15], there has been less investigation of how the distribution of admissions with respect to these characteristics has changed over time at the national level. Previous research for the UK has focused on trends in alcohol-related mortality [16–18]. Of the small evidence base, investigations have focused on either reporting overall trends in admissions [19] or trends in specific harms such as alcoholic liver disease [13, 20, 21] or alcohol-related poisonings [14]. Improving our understanding of trends in admissions at the national level will be key to informing national strategies aimed at reducing alcohol-related harms and narrowing social inequalities.

Our paper is timely given that England’s new guidelines on appropriate drinking levels have been altered to some of the lowest globally [22]. This is in part a response to England’s high levels of alcohol consumption, although when compared to other countries with similar levels of consumption England has higher rates of alcohol-related morbidities and (premature) mortality [10, 23, 24]. Despite England’s high levels of consumption, trends in consumption have also been falling since 2005 driven mostly by rising abstainers amongst younger adults [5]. Understanding whether trends in hospital admissions have followed trends in declining consumption is also important for identifying priority areas for strategies.

The aim of our study was to examine trends in alcohol-related admissions to hospital in England, with a focus on variations by sex, age and socioeconomic deprivation.

## Methods

### Data

Hospital Episode Statistics (HES) for hospital admissions in England for the 11-year period 2002/03 to 2013/14 (financial year 1st April to 31st March) were supplied by NHS Digital. HES data are routinely collected administrative data that record any hospital activity. Information extracted included the personal characteristics of each patient (age, sex, location of residence), along with up to 20 diagnoses. Data comprised individual ‘episodes’, each of which is a continuous period of care under a single consultant doctor during an admission.

We followed the approach taken by Public Health England, an executive agency of the Department of Health responsible for health promotion and protection, for identifying alcohol-related admissions [25]. Firstly, the data set was cleaned to address some known issues with HES. Only episodes that were finished, and were ‘ordinary’ (non-elective admissions or an elective admission expected to remain in hospital overnight), day case (elective admissions not requiring an overnight stay) or maternity admissions were included. We removed admissions with an age at the beginning of admission outside the range of 0 to 120, or where the sex was not recorded as male or female. We only considered admissions with an English postcode of residence so that our national estimates remained consistent with our estimates by level of deprivation, since the deprivation data were assigned using a patient’s residential address. This produced a small undercount of estimates as some admissions with unknown address or no fixed abode were alcohol-related (see Note 7 in Additional file 1: Table S1), but was necessary to allow for consistency in the data used throughout our results. The percentage of episodes which remained following cleaning over the study period was 89.72% (see Additional file 1: Table S1 for more details).

Alcohol-related conditions were weighted using Public Health England’s age group and sex specific population attributable fractions (PAFs) [25, 26]. PAFs represent the proportion of cases (i.e. hospital admissions) at the population level that might be attributed to an exposure (i.e. alcohol consumption). PAFs were age- and sex-specific, and were adjusted for alcohol consumption [26]. Negative PAFs, which suggest a protective effect of alcohol, were excluded. We identified alcohol-related admissions based on Public Health England’s ‘narrow’ measure. Our approach is defined in the paragraph below.

Each HES episode can contain up to 20 diagnoses, with the first diagnosis being the primary diagnosis (diagnoses were coded using ICD-10 throughout the study period). The narrow measure was calculated based on the primary diagnosis of an admission, with external conditions taken from secondary diagnostic positions 2 to 14 (external conditions, which are environmental causes of injury occurring outside the body, do not feature as the primary diagnosis). During the time period studied, the number of secondary diagnostic positions was expanded from 14 to 20, but we only considered up to 14 so that there was consistency throughout the period. Where there were multiple alcohol-related conditions recorded within an episode, we classified the admission using the condition with the largest PAF. If there were two conditions with the same PAF, we used the one from the lowest diagnostic position (i.e. closest to diagnosis position 1). An admission can comprise more than one episode of care if patients are transferred from the care of one consultant to another. However, 86.7% of all episodes only contained a single episode. We considered only the first episode from each admission.

We used four outcome measure categories of alcohol-related harm based on previous research [25–27]. These were (i) acute conditions wholly attributable to alcohol consumption, (ii) chronic conditions wholly attributable to alcohol consumption, (iii) acute conditions partially attributable to alcohol consumption, and (iv) chronic conditions partially attributable to alcohol consumption. Additional file 2: Table S2 presents which conditions were found in each category (based on Public Health England’s guidance). For acute conditions, we considered only emergency admissions. For chronic conditions, we combined emergency and non-emergency admissions.

In addition, we also examined four specific conditions which were wholly attributable to alcohol consumption, two acute and two chronic. These were the most common specific conditions within the acute and chronic wholly attributable to alcohol categories. The two acute conditions were (i) ‘Acute Intoxication subcategory of Mental and Behavioural Disorders due to use of Alcohol’ (ICD-10 code F10.0), and (ii) ‘Intentional self-poisoning due to alcohol’ (ICD-10 code X65). The two chronic conditions were (i) ‘All other Mental and Behavioural Disorders due to use of Alcohol’ (ICD-10 code F10.1-F10.9), and ‘Alcoholic Liver Disease’ (ICD-10 code K70).

We also calculated ‘non-alcohol’ related admissions to understand how our other measures compared to general trends in admissions. Non-alcohol related admissions were identified as any admission that contained a condition that had no known association to alcohol as defined using the population attributable fractions used previously (i.e. PAF = 0). We chose to exclude any condition with a partial association.

Counts of admissions were calculated for each outcome measure by Lower Super Output Area (LSOA). LSOAs are census areas created to disseminate administrative data and contain an average population size of approximately 1500 people. This allowed analysis by area-based socioeconomic deprivation. Annual mid-year Office for National Statistics (ONS) population estimates for LSOAs by five year age band and sex were used to calculate rates. We also attached the English Indices of Deprivation (IMD). IMD is a multi-dimensional neighbourhood-level indicator of socioeconomic deprivation for LSOAs [28]. We used the ‘income deprivation’ domain to avoid circularity issues since the overall index contains a health domain. Each measure of IMD was divided into categories based on quintiles of its level of income deprivation. IMD scores were assigned to HES records based on the closest year for which IMD scores were available (2004, 2007, and 2010). The 2015 index was not available at the time of analysis.

### Statistical analysis

We calculated the crude rate of admissions at the national level and age specific rates for five year age bands. The results were further disaggregated by quintile of deprivation, and we then calculated the directly standardised rate of admission for each quintile category to account for differing age structures between categories (age-specific rates were used for analysing trends by age). 95% confidence intervals were calculated for estimates using Byar’s methodology [25]. All analyses were stratified by sex. We visualised our estimates using line plots and Lexis surfaces. Lexis surfaces allow for the simple visualisation of complex patterns through the plotting of admission rates by age band and year together.

## Results

Table 1 presents descriptive statistics of admissions over the study period. Total admissions for any condition attributable to alcohol (i.e. the sum of our primary outcomes) in England increased from 201,398 in 2002/03 to 303,716 in 2013/14. The relative increase in the number of admissions attributable to alcohol over the study period (1.51 times larger in 2013/14) was larger than the relative increase for overall non-alcohol attributable admissions (1.39 times larger in 2013/14) even following disaggregating between emergency and non-emergency admissions. The largest relative difference was for acute conditions wholly attributable to alcohol (2.26 times larger in 2013/14), with a doubling of admissions for each specific acute condition measured.

**Table 1:** Number of admissions to hospital for alcohol-associated conditions at the start (2002/03) and end (2013/14) of the study period for England

		2002/03	2013/14	Relative Change
Condition categories	Wholly Acute	23,172	52,291	2.26
	Wholly Chronic	34,159	50,142	1.47
	Partially Acute	44,589	63,671	1.43
	Partially Chronic	99,478	137,612	1.38
Specific conditions wholly attributable to alcohol	Acute Intoxication subcategory of Mental and Behavioural Disorders due to use of Alcohol (F10.0)	7013	15,546	2.22
	All Other Mental and Behavioural Disorders due to use of Alcohol (F10.1-F10.9)	19,375	26,921	1.39
	Alcoholic Liver Disease (K70)	11,135	16,142	1.45
	Intentional self-poisoning due to alcohol (X65)	11,966	31,232	2.61
Total alcohol-related admissions	Total Admissions	201,398	303,716	1.51
	Crude Rate (per 100,000)	402	560	1.39
Total non-alcohol related admissions	Total Admissions	9,450,741	13,095,242	1.39
	Total Emergency Admissions	3,052,094	4,071,187	1.33
	Total Non-Emergency Admissions	6,398,647	9,024,055	1.41
Population (n)		50,141,285	54,276,638	1.08

Partially attributable condition totals are a sum of fractions of admissions for conditions which are partially attributable to alcohol

**Fig. 1:**
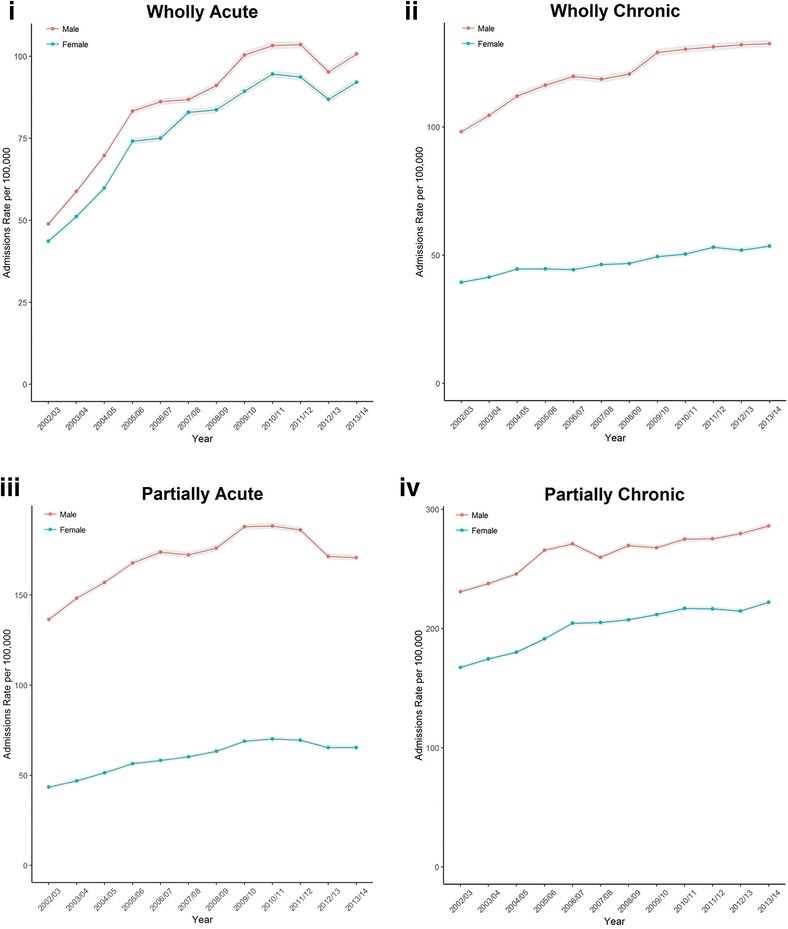
Crude rate per 100,000 (with shaded 95% Confidence Intervals) of hospital admissions by sex for England, 2002/03 to 2013/14: (**i**) acute conditions wholly attributable to alcohol (titled: ‘Wholly Acute’), (**ii**) chronic conditions wholly attributable to alcohol (‘Wholly Chronic’), (**iii**) acute conditions partially attributable to alcohol (‘Partially Acute’), (**iv**) chronic conditions partially attributable to alcohol (‘Partially Chronic’)

Figure 1 plots the crude rate over the study period for our four main outcome variables (acute and chronic conditions wholly attributable to alcohol, and acute and chronic conditions partially attributable to alcohol). Admission rates increased over the study period for each measure. There was a sharp increase in acute conditions wholly attributable to alcohol particularly between 2002/03 and 2005/06. Admission rates were higher for males compared to females for each measure, and trends were fairly consistent by sex.

**Fig. 2:**
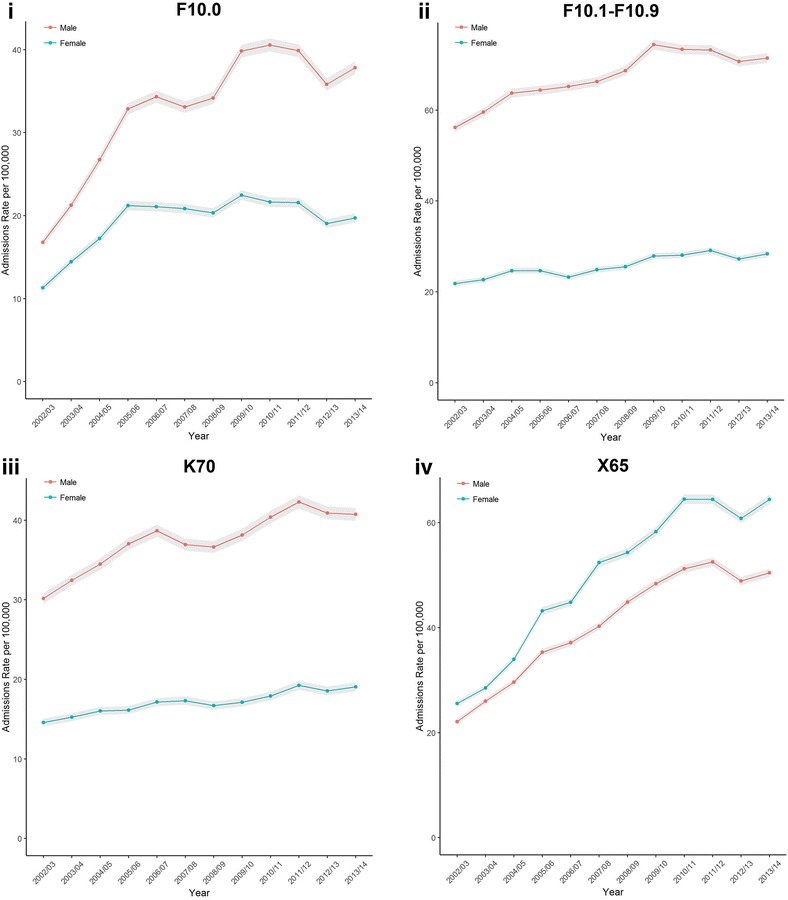
Crude rate per 100,000 (with shaded 95% Confidence Intervals) of hospital admissions by sex for England, 2002/03 to 2013/14: (**i**) Acute Intoxication subcategory of Mental and Behavioural Disorders due to use of Alcohol (titled: ‘F10.0’)’, (**ii**) All other Mental and Behavioural Disorders due to use of Alcohol (‘F10.1-F10.9′), (**iii**) Alcoholic Liver Disease (‘K70′), (**iv**) Intentional self-poisoning due to alcohol (‘X65’)

Focusing on four specific conditions that were wholly attributable to alcohol (two acute and two chronic) revealed greater variability in admission trends (Fig. 2). The two chronic conditions (‘All other Mental and Behavioural Disorders due to use of Alcohol’ and ‘Alcoholic Liver Disease’) displayed similar trends with increasing admission rates (although admission rates were smaller for ‘Alcoholic Liver Disease’). ‘Acute Intoxication subcategory of Mental and Behavioural Disorders due to use of Alcohol’ admission rates increased over the study period for both males and females. The majority of the overall increase for females occurred by 2005/06 with trends levelling off and declining slightly afterwards. There was also a sharp increase up to 2005/06 for males, and while the rate of the increase slowed down after this period admission rates continue rising unlike for females. Admission rates for ‘Intentional self-poisoning due to alcohol’ were the only specific condition where admission rates were higher for females. There were a sharp increase in admissions for the condition over the study period for both males and females, with trends increasing larger for females resulting in a widening gap between sexes by the end of the period.

**Fig. 3:**
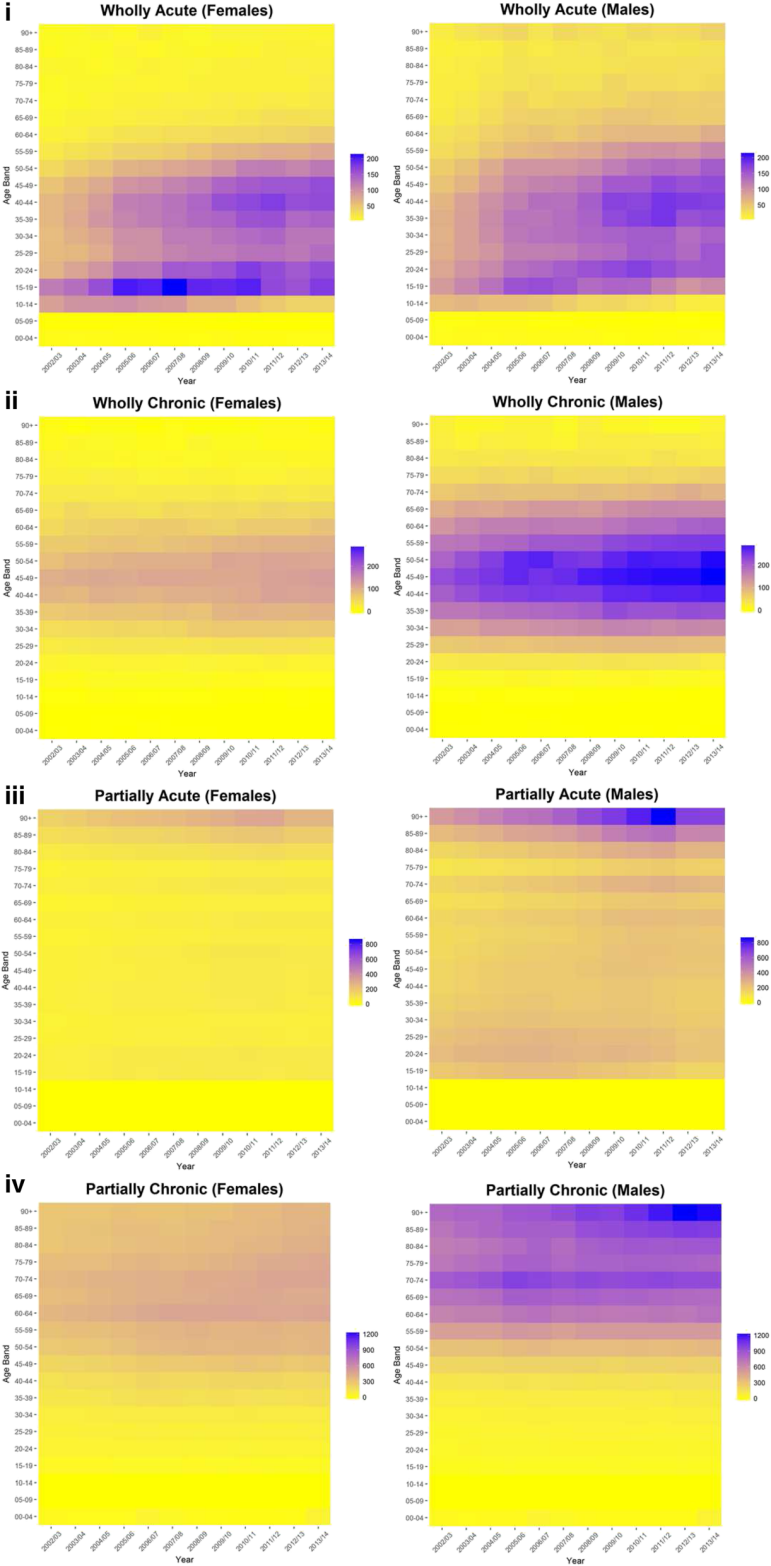
Age-specific (5-year age bands) rate per 100,000 of hospital admissions by sex in England, 2002/03 to 2013/14: (**i**) acute conditions wholly attributable to alcohol (titled: ‘Wholly Acute’), (**ii**) chronic conditions wholly attributable to alcohol (‘Wholly Chronic’), (**iii**) acute conditions partially attributable to alcohol (‘Partially Acute’), (**iv**) chronic conditions partially attributable to alcohol (‘Partially Chronic’) Note: Scales are same for each sex, but vary by measure.

Figures 3 presents the results from stratifying our condition categories by age. Acute conditions wholly attributable to alcohol demonstrated a bimodal pattern with respect to age that was similar for males and females, with higher rates in middle age (35–54) and adolescence/young adults (15–24). The bimodal distribution was sharpest for females who had the highest admission rates observed for individuals aged 15–19 for the middle part of the study period. Admission rates increased progressively over our study period for the middle age group (for both females and males). These trends differed to individuals aged 15–19 which peaked around the middle of the study period before declining (for both males and females). Trends for individuals aged 20–24 declined following 2010/11. Chronic conditions wholly attributable to alcohol were concentrated in middle aged men (especially individuals aged 40–54), with admission rates also increasing over time for this age group as well (a similar trend is observed for females, albeit at smaller admission rates). Admission rates for both acute and chronic conditions partially attributable to alcohol increased with age, and were both higher for males.

**Fig. 4:**
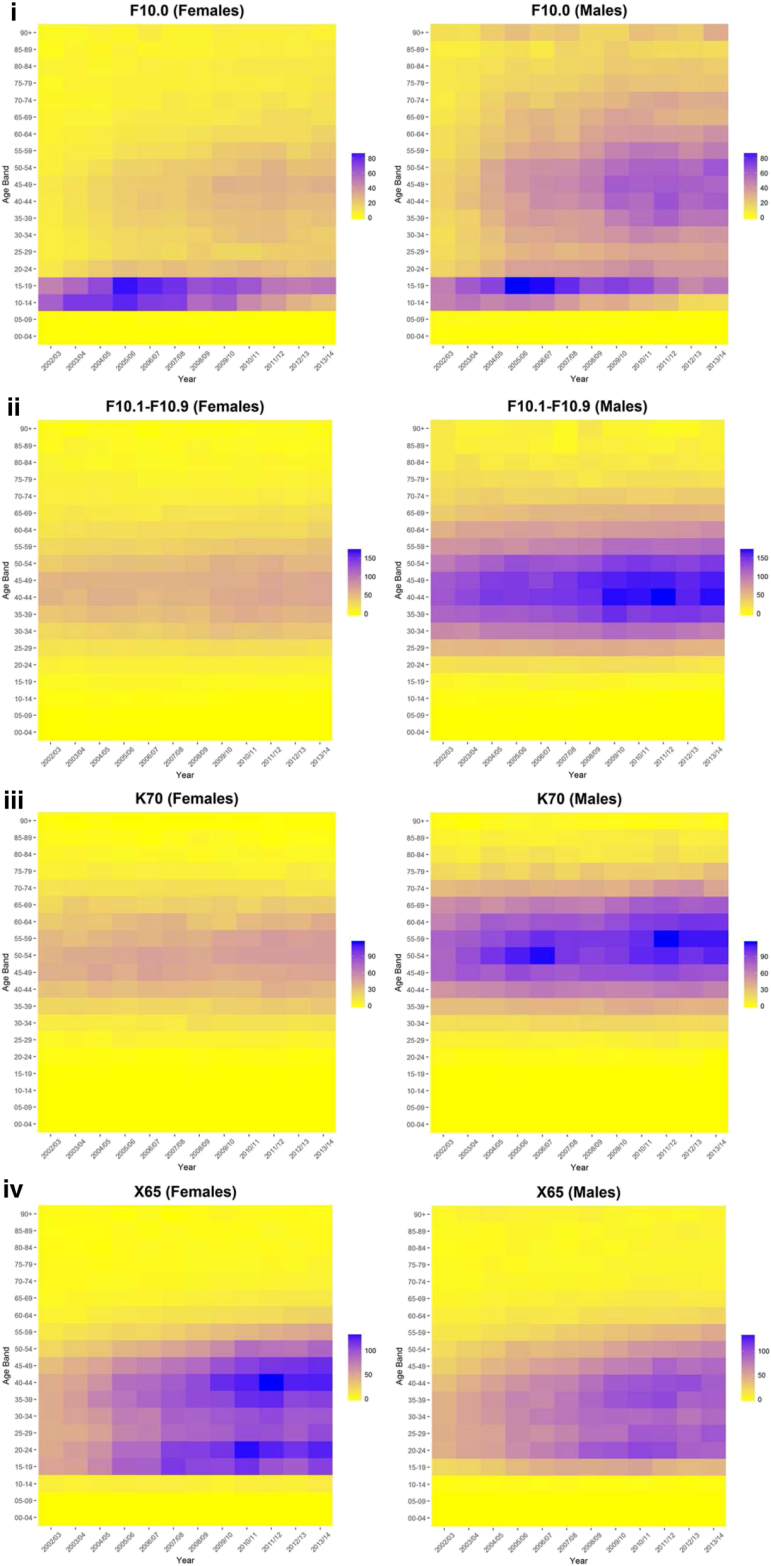
Age-specific (5-year age bands) rate per 100,000 of hospital admissions by sex in England, 2002/03 to 2013/14: (**i**) Acute Intoxication subcategory of Mental and Behavioural Disorders due to use of Alcohol (titled: ‘F10.0’)’, (**ii**) All other Mental and Behavioural Disorders due to use of Alcohol (‘F10.1-F10.9′), (**iii**) Alcoholic Liver Disease (‘K70′), (**iv**) Intentional self-poisoning due to alcohol (‘X65’). Note: Scales are same for each sex, but vary by condition.

Exploring the specific conditions wholly attributable to alcohol helps to explain the trends seen for all acute conditions wholly attributable to alcohol combined (Fig. 4). Admission rates for ‘Acute Intoxication subcategory of Mental and Behavioural Disorders due to use of Alcohol’ were highest for the age group 10–19 for both males and females. Admission rates for individuals aged 15–19 followed similar patterns for males and females, but for individuals aged 10–14 admission rates were higher for females. There were also increasing admission rates for middle aged males over study period contrary to trends for younger ages. ‘Intentional self-poisoning due to alcohol’ displayed a bimodal distribution by age with higher rates for middle-aged individuals (35–49) and young adults [20–24]. The bimodal distribution was more pronounced for females compared to males. Trends at each of these age groups were similar, having increased over the study period. The two specific conditions wholly attributable to alcohol (‘All other Mental and Behavioural Disorders due to use of Alcohol’ and ‘Alcoholic Liver Disease’) experienced little change over the study period for females (and were considerably lower than compared to males). Admission rates were higher among older age groups for ‘Alcoholic Liver Disease’ (50–59), being concentrated among the age group 40–49 for ‘All other Mental and Behavioural Disorders due to use of Alcohol’. Both conditions saw increases in admission rates over the study period (particularly in those age groups).

**Fig. 5:**
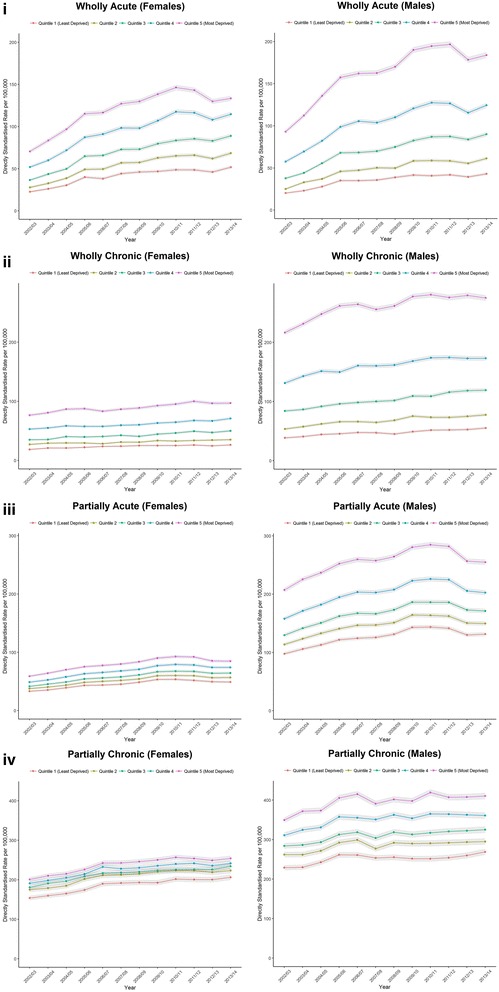
Directly standardised rate of hospital admissions for England split by deprivation quintile: (**i**) acute conditions wholly attributable to alcohol (titled: ‘Wholly Acute’), (**ii**) chronic conditions wholly attributable to alcohol (‘Wholly Chronic’), (**iii**) acute conditions partially attributable to alcohol (‘Partially Acute’), (**iv**) chronic conditions partially attributable to alcohol (‘Partially Chronic’).

We repeated our analyses by deprivation category to understand how trends contributed to social inequalities. Across each of our condition categories, admission rates were higher in the most deprived areas compared to the least deprived areas (Fig. 5). The gap between the most and least deprived areas was wider for males for each measure. For acute and chronic conditions partially attributable to alcohol, there difference between the most and least deprived areas remained mostly consistent over the study period. These trends were contrary to acute and chronic conditions wholly attributable to alcohol where trends mainly diverged. Differences between the most and least deprived areas were most pronounced for females in acute conditions wholly attributable to alcohol. While admission rates for males were typically higher than compared to females, admission rates for acute conditions wholly attributable to alcohol in the least deprived areas where higher for females.

**Fig. 6:**
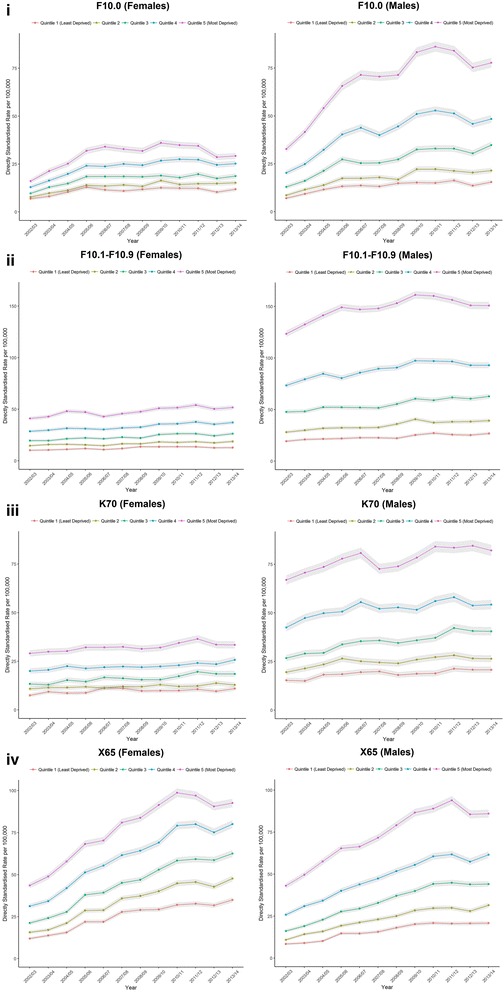
Directly standardised rate of hospital admissions for England of four conditions wholly attributable to alcohol: (**i**) Acute Intoxication subcategory of Mental and Behavioural Disorders due to use of Alcohol (titled: ‘F10.0’)’, (**ii**) All other Mental and Behavioural Disorders due to use of Alcohol (‘F10.1-F10.9′), (**iii**) Alcoholic Liver Disease (‘K70’), (**iv**) Intentional self-poisoning due to alcohol (‘X65’). Note: Scales are same for each sex, but vary by condition

Figure 6 presents trends by deprivation category for the specific conditions. Both of the chronic conditions (‘All other Mental and Behavioural Disorders due to use of Alcohol’ and ‘Alcoholic Liver Disease’ remained fairly flat over time for females compared to males where there were increases in the most deprived areas. ‘Acute Intoxication subcategory of Mental and Behavioural Disorders due to use of Alcohol’ saw large increases in admission rates in the most deprived areas for males, with less pronounced increases in less deprived areas. For females, there was a small increase at the start of the period for each category, before admission rates levelled off. The difference in admission rates between the most and least deprived areas were widest for males for each condition other than ‘Intentional self-poisoning due to alcohol’. Intentional self-poisoning due to alcohol was the only measure where admission rates for each deprivation category were higher for females (in 2013/14).

## Discussion

### Key results

Our study presents population-level trends in hospital admissions attributable to alcohol by age, sex and level of socioeconomic deprivation. Total admissions attributable to alcohol increased from 201,398 in 2002/03 to 303,716 in 2013/14. While these increases have occurred during a period of overall increases in non-alcohol attributable admissions, the relative increase in alcohol-attributable admissions was larger than increases in overall, emergency and non-emergency non-alcohol attributable admissions. The largest relative increase in admission type was for acute admissions wholly attributable to alcohol which doubled. Admission rates were consistently higher for males compared to females across all of our outcome measures other than ‘Intentional self-poisoning due to alcohol’ which has more than doubled for both males and females over our study period.

Chronic admissions wholly attributable to alcohol were concentrated in middle age, whereas for acute admissions wholly attributable to alcohol there was a clear bimodal distribution (although admission rates in adolescents/young adults declined over the period). Both acute and chronic partially attributable admissions were higher in the oldest ages. However, the concentrated in middle aged males resulted that in 2013/14 men aged between 35 and 54 consisted 27.4% of the male population but accounted for 34% of all male admissions attributable to alcohol, 44.5% of all male acute admissions wholly attributable to alcohol and 56.4% of all male chronic admissions wholly attributable to alcohol.

Finally, we observe a social gradient with admission rates higher in the most deprived areas compared to the least deprived areas. Social inequalities were wider for males compared to females, and they became wider over the study period particularly for acute conditions wholly attributable to alcohol. ‘Intentional self-poisoning due to alcohol’ was the only condition where admission rates were higher for females across each deprivation category.

### Limitations

While HES is routinely collected data on all hospital admissions across England, there are multiple issues with the data which limit the interpretation of our observations. We do not include A&E attendances in our data (only if an individual was admitted) which may lead to an undercount of the harms associated with alcohol. There may be individuals who do not seek medical care and if over the period these individuals have changed their behaviours through seeking care it may affect our observed trends. The trends we observe may be partly explained by changes over time in the quality of HES. Changes in coding practice over time could partly influence our observed trends. In 2003/04, ‘Payment by Results’ (PbR) was introduced whereby hospitals were paid for fully reporting all treatments provided to patients [29, 30]. Increasing admission rates may partly reflect such financial incentives which capture more information which previously was missed. While we only use the primary diagnosis for most alcohol-related conditions, PbR has been associated with increasing depth of secondary diagnoses which may influence trends for external conditions. These changes have also occurred alongside rising levels of overall admissions which could influence our trends ([29]; also see Additional file 1: Table S1). However, increases in alcohol-attributable admissions were larger than increases in admissions for non-alcohol-attributable admissions (including split by emergency and non-emergency admissions).

Missing data was also an issue particularly as it could not always be ascertained that it was not missing randomly (i.e. see Additional file 1: Table S1 Note 7) which may introduce bias into our results (although the level of bias is likely to be small). There have also been increasing usage of ‘bucket codes’, which are diagnoses whereby the cause is unknown and therefore represent missing data, for emergency admissions over the same period (Additional file 3: Figure S1). The use of bucket codes may produce an undercount of admission rates. Diagnostic accuracy is also important with Burns et al. [30] estimating that 80% of primary diagnoses are correctly coded.

The partially attributable conditions used in calculating our measures are based on PAFs that assume a causal relationship between alcohol and each condition [26]. Whilst PAFs are useful for estimating population level patterns, we do not know which individual admissions were due to alcohol. Incorrectly attributing non-alcohol associated admissions to our partially-attributable estimates may introduce error into our results. This is evident in our age-specific analyses for partially attributable conditions that suggest greater alcohol-related harm in the elderly, which is mostly driven by the higher rates of some partially-attributable conditions at old age despite their small PAFs (e.g. falls). The issue is also problematic when analysing trends since factors unrelated to alcohol may be driving changes over time [31]. We therefore suggest interpreting our results for conditions partially attributable to alcohol cautiously.

We used the narrow measure of alcohol-related admissions, which only consider the primary diagnosis to ascertain admissions for non-external conditions [25]. While this approach may underestimate alcohol-related admissions, particularly wholly attributable conditions found in the secondary diagnostic positions that are not evident in the primary diagnosis, the measure is less sensitive to changes in coding practice over time (e.g. increased usage of secondary diagnoses over the period). We also did not investigate trends in case severity of admissions, which may again lead to an underestimate of alcohol-related admissions.

Whilst we focussed on hospital admissions as our measure of alcohol-related harm, our estimates included multiple repeat admissions of individuals which may have overestimated the scale of the problem if we are interested in understanding individual-level patterns. However, admissions represent the actual demand for hospitals and therefore are important for understanding the total pressure on health services.

### Interpretation

Increasing alcohol-attributable admissions have been noted for England both overall [19] and for specific conditions [13, 14, 20, 21]. Our study builds on this small evidence-base by presenting a detailed investigation of how trends in alcohol-attributable admissions vary by sex, age and socioeconomic deprivation. Our results also corroborate with experiences in other countries [32–35].

The increasing burden of alcohol-attributable admissions placed on the NHS, combined with increasing pressures on scarce healthcare resources; suggest the need for increased focus on preventive measures. Our results suggest that the optimum intervention may differ by population sub-group, depending on age, sex and level of deprivation. The scale of the problem combined with the high costs involved in treating alcohol-attributable harms (as well as the wider costs to society) suggest that population-level preventive measures could be cost effective [10].

Recent declines in total alcohol consumption experienced at the national level since 2005 have not translated into downward trends in alcohol-related hospital admissions [5, 10]. Similar diverging trends of consumption and hospital admissions have been reported elsewhere [31]. There are multiple explanations for this. There may be a time lag effect and the effects of declining consumption have yet to result in fewer admissions. This would be a stronger explanation for trends in chronic conditions compared to acute. Whilst declining consumption appears to be driven by declines amongst younger adults, we only see some evidence for this in admissions due to ‘Acute Intoxication subcategory of Mental and Behavioural Disorders due to use of Alcohol’ [5]. It may also be that under-reporting bias in consumption is increasing over time [36]. Obtaining reliable self-reported survey information on alcohol consumption is difficult. Finally, it may be that consumption is declining faster in those individuals at lower risk of admission, and in those at high risk of admission consumption may be increasing [10]. Trends in alcohol-related mortality rates have, however, followed consumption trends more closely [37]. Understanding the disparity between morbidity and mortality trends, and how they relate to trends in consumption will be an important direction for future research.

While admission rates were higher for males for most conditions and ages, we found higher rates of ‘Intentional self-poisoning due to alcohol’ in females, particularly younger females. This finding follows increases in overall levels in self-poisoning and self-harm amongst females in England [38, 39]. Increased ‘felt’ pressures on younger women may be leading to greater self-harm through alcohol [40–42]. Our figures may also underestimate the scale of the issue since not all individuals who self-harm will be admitted to hospital [43]. However, it also follows trends that self-harm cases are now more likely to be admitted to hospital [44]. Whilst alcoholic liver disease has received much attention in the literature [10, 13, 20, 21, 45], there is clear need for greater investigation of the wider health-related harms attributable to alcohol.

Our results demonstrate wide social inequalities in alcohol-attributable admissions, supporting evidence from elsewhere [4, 8, 9, 11, 12]. There is a clear social gradient in admissions for each outcome measure, and inequalities were widest for middle aged males. The social gradient exists despite similar levels of consumption in deprived and affluent areas; termed the ‘alcohol harm paradox’. Bellis and colleagues offer two possible explanations for the paradox [11]. Firstly, individuals from deprived areas tend to engage in multiple risky behaviours (e.g. smoking or unhealthy diets), which interact with alcohol consumption to result in a greater risk of hospitalisation. Secondly, individuals in deprived areas engage in different drinking behaviours (e.g. binge drinking) despite similar overall levels of consumption. Siegler and colleagues also suggest that social inequalities in harms may be influenced by ‘social drift’, whereby heavy drinkers move down the social gradient due to the harms of their consumption, although there is less evidence for this [8]. Given that many of these alcohol-attributable admissions are preventable, this represents a key policy area to reduce overall social inequalities in health.

## Conclusions

Our study produces the first evidence of population trends in alcohol-attributable hospital admissions by age, sex and level of socioeconomic deprivation by condition for England. While our results need to be interpreted within the context of increasing hospital admissions and improved data recording (as well as other limitations in the data quality), they present a stark picture of increasing alcohol-attributable harms. There have been large relative increases in alcohol-attributable admissions particularly for acute conditions wholly attributable to alcohol. Many of these increases have become concentrated in middle-aged males and in deprived areas, and these represent continued key areas for policy officials. Of concern are the large increases in young females being admitted to hospital for ‘Intentional self-poisoning due to alcohol’ a finding largely uncovered in the literature. Given the large associated costs of alcohol-related harms in England, understanding population level trends is an important step to be able to develop effective strategies to tackle these trends.

## Additional files

Additional file 1: Table S1. Information on the number of episodes at each stage of the data cleaning process for each year of HES data. (DOCX 20.0 kb)

Additional file 2: Table S2. List of conditions by outcome measure. (DOCX 21 kb)

Additional file 3: Figure S1. Number of episodes with a primary diagnoses containing an ICD-10 code of R00-R99 (‘Symptoms, signs and abnormal clinical and laboratory findings, not elsewhere classified’). (DOCX 17 kb)

## Abbreviations

HES: Hospital Episode Statistics
HSCIC: Health and Social Care Information Centre
IMD: English Indices of Deprivation
LSOA: Lower Super Output Area
NHS: National Health Service
PAF: Population Attributable Fraction
