# Supplementary material for: Trends in alcohol-related admissions to hospital by age, sex and socioeconomic deprivation in England, 2002/03 to 2013/14
Source: BMC Public Health. 2017 May 8;17:412. doi: 10.1186/s12889-017-4265-0 (PMC5423017; doi:10.1186/s12889-017-4265-0)
Supplement: Supplementary file 1 — Information on the number of episodes at each stage of the data cleaning process for each year of HES data. (DOCX 20.0 kb) [file 12889_2017_4265_MOESM1_ESM.docx]

| **Steps:** | **HES Year** | | | | | | | | | | | |
| --- | --- | --- | --- | --- | --- | --- | --- | --- | --- | --- | --- | --- |
|  | 2002/03 | 2003/04 | 2004/05 | 2005/06 | 2006/07 | 2007/08 | 2008/09 | 2009/10 | 2010/11 | 2011/12 | 2012/13 | 2013/14 |
| 1. Input Data | 13442308 | 14129373 | 14546126 | 15395157 | 15803643 | 16456185 | 17434446 | 18126831 | 18696242 | 18889329 | 19112187 | 19578568 |
| 2. Duplicate episodes removed | 53760 | 35753 | 39763 | 27474 | 51380 | 47916 | 37840 | 29884 | 27546 | 25933 | 28111 | 28049 |
| 3. Episodes that are finished | 13283455 | 13980604 | 14426236 | 15272330 | 15726871 | 16387426 | 17373923 | 18070329 | 18645762 | 18836541 | 19061207 | 19532135 |
| 4. Episodes that are an ordinary admission, day case or maternity | 12670116 | 13269039 | 13676325 | 14402161 | 14735462 | 15315742 | 16197459 | 16778486 | 17245679 | 17441771 | 17690435 | 18141734 |
| 5. Episodes with age between 0 and 120 | 12633937 | 13237628 | 13648235 | 14373587 | 14707971 | 15283021 | 16136912 | 16725534 | 17186604 | 17368029 | 17603882 | 18043558 |
| 6. Episodes with males or females | 12632427 | 13236159 | 13646585 | 14371667 | 14701353 | 15275470 | 16135218 | 16721073 | 17184004 | 17365747 | 17602015 | 18041642 |
| 7. Episodes that are England only | 12086231 | 12676997 | 13053531 | 13748488 | 14340330 | 14776072 | 15799767 | 16410382 | 16866803 | 16562687 | 16814060 | 17744131 |
| Overall percentage change | 89.91 | 89.72 | 89.74 | 89.30 | 90.74 | 89.79 | 90.62 | 90.53 | 90.21 | 87.68 | 87.98 | 90.63 |

**Additional file 1: Table S1.** Information on the number of episodes at each stage of the data cleaning process for each year of HES data.

Note: The data cleaning process involved the following steps:

1. Reading in the data provided by NHS Digital. The number of episodes were then checked against those provided by NHS Digital and were all found to be the same.
2. We removed all episodes that we determined were duplicates. Duplicates were defined as two episodes which had the same; HES ID (a unique identifier for each individual), date of admission, date of discharge, episode order (i.e. the first episode of an admission is 1 and then any further episode within an admission sees this number increase sequentially) and the first recorded diagnosis (i.e. primary diagnosis). If a duplicate was found, we removed the episode which had the lowest value of the variable ‘epikey’. ‘Epikey’ is a unique identifier for each episode and is hierarchical so that a higher value represents an episode with more information (i.e. more recently entered into the system). The number of duplicates varies considerably by year, although declines over time and becomes more consistent in recent years.
3. We only considered episodes in the data that were finished.
4. Only admissions which were ordinary admissions, day case or maternity admissions were retained in the data set.
5. We kept all episodes where the age when an individual was admitted was between 0 and 120. There were no individuals with ages outside of these bounds, however there were some missing data removed.
6. We removed any episode that did not contain either males or females.
7. Finally, we retained all episodes where individuals contained an address which was located in England. Some individuals and these were usually individuals who lived on the borders or had travelled to England (e.g. on holiday) and were admitted. Individuals whose geographical area was ‘unknown’ or ‘no known fixed abode’ were also removed. The decision differs to Public Health England’s (PHE) approach for calculating alcohol-related admissions since they include these admissions in their estimates of national estimates. Since our study analysed differences in hospital admissions by level of neighbourhood deprivation (measured using an individual’s postcode of residence), we removed episodes with missing geographical data so that the data was consistent across each of our analyses. However, an issue with leaving these admissions out of the data was that some admissions with missing geographical codes were alcohol-related admissions (although Supplementary Table 1 demonstrates that these were only small numbers, this does introduce a small amount of undercounting when talking about patterns at the national level). Examining the primary diagnosis (variable ‘diag_01’) in episodes with missing geographical data, 5.3% of episodes with a diagnosis of ‘Toxic effect of alcohol’ (ICD code T51), 7.3% with ‘Mental and behavioural disorders due to use of alcohol’ (F10) and 1.9% with ‘Alcoholic Liver Disease’ (K70). 14.1% of all episodes (17% of admissions) with missing geographical data had a primary diagnosis of P05 or P07 (‘Disorders related to length of gestation and fetal growth’).
