# Supplementary material for: Trends in alcohol-related admissions to hospital by age, sex and socioeconomic deprivation in England, 2002/03 to 2013/14
Source: BMC Public Health. 2017 May 8;17:412. doi: 10.1186/s12889-017-4265-0 (PMC5423017; doi:10.1186/s12889-017-4265-0)
Supplement: Supplementary file 2 — List of conditions by outcome measure. (DOCX 21 kb) [file 12889_2017_4265_MOESM2_ESM.docx]

|  | **ICD-10 Code** | **Description** | |
| --- | --- | --- | --- |
|  |  |  | |
| **Conditions wholly attributable to alcohol - Acute conditions** | | | |
|  | F10.0 | Acute intoxication subcategory of mental and behavioural disorders due to use of alcohol | |
|  | R78.0 | Excess alcohol blood levels | |
|  | T51 | Toxic effect of alcohol | |
|  | X45 | Accidental poisoning by and exposure to alcohol | |
|  | X65 | Intentional self-poisoning by and exposure to alcohol | |
|  | Y15 | Poisoning by and exposure to alcohol, undetermined intent | |
|  | Y90 | Evidence of alcohol involvement determined by blood alcohol level | |
|  | Y91 | Evidence of alcohol involvement determined by level of intoxication | |
|  | | | |
| **Conditions wholly attributable to alcohol - Chronic conditions** | | | |
|  | E24.4 | Alcohol-induced pseudo-Cushing’s syndrome | |
|  | F10.1-F10.9 | All other mental and behavioural disorders due to use of alcohol | |
|  | G31.2 | Degeneration of nervous system due to alcohol | |
|  | G62.1 | Alcoholic polyneuropathy | |
|  | G72.1 | Alcoholic myopathy | |
|  | I42.6 | Alcoholic cardiomyopathy | |
|  | K29.2 | Alcoholic gastritis | |
|  | K70 | Alcoholic liver disease | |
|  | K85.2 | Alcohol-induced acute pancreatitis | |
|  | K86.0 | Alcohol-induced chronic pancreatitis | |
|  | Q86.0 | Fetal alcohol syndrome (dysmorphic) | |
|  |  |  | |
| **Conditions partially attributable to alcohol - Chronic conditions** | | | |
|  | **Infectious and parasitic diseases** | | |
|  | A15-A19 | Tuberculosis | |
|  | **Cancer** |  | |
|  | C00-C14 | Lip, oral cavity and pharynx | |
|  | C15 | Oesophagus | |
|  | C18-C20, C21 | Colorectal | |
|  | C22 | Liver and intrahepatic bile ducts | |
|  | C32 | Larynx | |
|  | C50 | Breast | |
|  | **Diseases of the nervous system** | | |
|  | G40-G41 | Epilepsy and Status epilepticus | |
|  | **Cardiovascular disease** | | |
|  | I10-I15 | Hypertensive diseases | |
|  | I47-I48 | Cardiac arrhythmias | |
|  | I60-I62, I69.0-I69.2 | Haemorrhagic stroke | |
|  | I63-I66, I69.3-I69.4 | Ischaemic stroke | |
|  | I85 | Oesophageal varices | |
|  | **Respiratory infections** | | |
|  | J10-J11(.0), J12-J15, J18 | Pneumonia | |
|  | **Digestive disease** | | |
|  | K73, K74 | Unspecified liver disease | |
|  | K85, K861 (except K852) | Acute and chronic pancreatitis | |
|  | **Pregnancy and childbirth** | | |
|  | O03 | Spontaneous abortion | |
|  | P05-P07 | Low birth weight | |
|  |  |  | |
| **Conditions partially attributable to alcohol - Acute conditions** | | | |
|  | **Unintentional injuries** | | |
|  | § | Road/Ped traffic accidents | |
|  | X40–X49 (except X45) | Poisoning | |
|  | W00-W19 | Fall injuries | |
|  | X00-X09 | Fire injuries | |
|  | W65-W74 | Drowning | |
|  | §§ | Other unintentional injuries | |
|  | **Intentional injuries** | | |
|  | X60-X84, Y87.0 (excl X65) | Intentional self-harm | |
|  | Y10-Y34, Y872 (except Y15) | Event of undetermined intent | |
|  | X85-Y09, Y87.1 | Assault | |
| § = V021-V029, V031-V039, V041-V049, V092, V093, V123-V129, V133-V139, V143-V149, V194-V196, V203-V209, V213-V219, V223-V229, V233-V239, V243-V249, V253-V259, V263-V269, V273-V279, V283-V289, V294-V299, V304-V309, V314-V319, V324-V329, V334-V339, V344-V349, V354-V359, V364-V369, V374-V379, V384-V389, V394-V399, V404-V409, V414-V419, V424-V429, V434-V439, V444-V449, V454-V459, V464-V469, V474-V479, V484-V489, V494-V499, V504-V509, V514-V519, V524-V529, V534-V539, V544-V549, V554-V559, V564-V569, V574-V579, V584-V589, V594-V599, V604-V609, V614-V619, V624-V629, V634-V639, V644-V649, V654-V659, V664-V669, V674-V679, V684-V689, V694-V699, V704-V709, V714-V719, V724-V729, V734-V739, V744-V749, V754-V759, V764-V769, V774-V779, V784-V789, V794-V799, V803-V805, V811, V821, V830-V833, V840-V843, V850-V853, V860-V863, V870-V878, V892. | | |  |
| §§ = V01, V090, V091, V099, V100-V109, V110-V119, V120-122, V130-132, V140-V142, V150-V159, V160-V169, V170-V179, V180-V189, V191-V193, V20-V28: 0.1–0.2; V290-V293, V30-V38: 0.1–0.2; V390-V393, V40-V48: 0.1–0.2; V490-V493, V50-V58: 0.1–0.2; V590-V593, V60-V68: 0.1–0.2; V690-V693, V70-V78: 0.1–0.2; V790-V793, V800, V801, V806–V809, V810, V812–V819, V820, V822–V829, V834–V839, V844–V849, V854–V859, V864–V869, V879, V88, V890, V891, V893–V899, V90-V94, V95-V97, V98-V99, W20-W52, W75-W84, W85–W99, X10-X19, X20-X29, X30-X33, X50-X57, X58, X59, Y40-Y84 Y85, Y86, Y88, Y89. | | |  |

**Additional file 2: Table S2.** List of conditions by outcome measure.
