# Supplementary material for: Trends in alcohol-related admissions to hospital by age, sex and socioeconomic deprivation in England, 2002/03 to 2013/14
Source: BMC Public Health. 2017 May 8;17:412. doi: 10.1186/s12889-017-4265-0 (PMC5423017; doi:10.1186/s12889-017-4265-0)
Supplement: Supplementary file 3 — Number of episodes with a primary diagnoses containing an ICD-10 code of R00-R99 (‘Symptoms, signs and abnormal clinical and laboratory findings, not elsewhere classified’). (DOCX 17 kb) [file 12889_2017_4265_MOESM3_ESM.docx]

**Additional file 3: Figure S1**. Number of episodes with a primary diagnoses containing an ICD-10 code of R00-R99 (‘Symptoms, signs and abnormal clinical and laboratory findings, not elsewhere classified’).
